# Epitope mapping *via in vitro* deep mutational scanning methods and its applications

**DOI:** 10.1016/j.jbc.2024.108072

**Published:** 2024-12-14

**Authors:** Meredith M. Keen, Alasdair D. Keith, Eric A. Ortlund

**Affiliations:** Department of Biochemistry, Emory School of Medicine, Emory University, Atlanta, Georgia, USA

**Keywords:** epitope mapping, deep mutational scanning, linking genotype to phenotype, antibody engineering, viral surveillance, immunotherapies, diagnostics, vaccine design

## Abstract

Epitope mapping is a technique employed to define the region of an antigen that elicits an immune response, providing crucial insight into the structural architecture of the antigen as well as epitope-paratope interactions. With this breadth of knowledge, immunotherapies, diagnostics, and vaccines are being developed with a rational and data-supported design. Traditional epitope mapping methods are laborious, time-intensive, and often lack the ability to screen proteins in a high-throughput manner or provide high resolution. Deep mutational scanning (DMS), however, is revolutionizing the field as it can screen all possible single amino acid mutations and provide an efficient and high-throughput way to infer the structures of both linear and three-dimensional epitopes with high resolution. Currently, more than 50 publications take this approach to efficiently identify enhancing or escaping mutations, with many then employing this information to rapidly develop broadly neutralizing antibodies, T-cell immunotherapies, vaccine platforms, or diagnostics. We provide a comprehensive review of the approaches to accomplish epitope mapping while also providing a summation of the development of DMS technology and its impactful applications.

Epitope mapping, employed to identify the epitope of an antigen, has been used in the development of therapeutics and the expansion of basic scientific knowledge. Epitopes generally consist of 8 to 26 amino acids; 2 to 5 of these residues are identified as the interaction “hotspot” with the antigen binding site, also known as the paratope, of a protein that is generally involved in the adaptive immune system (1, 2, 3). Structural analysis of the epitope provides key insight used in the rational design of immunotherapies. Moreover, protection against antigenic sin—a phenomenon wherein the immune system fails to mount a secondary exposure due to variation in the epitope from the initial exposure—is accomplished using the insights from epitope mapping, which helps to inform the rational development of broadly protecting immunotherapies (3). Traditional means of epitope mapping like X-ray crystallography, Cryo-EM, and NMR provide high-resolution structures; however, this is a low-throughput and laborious approach considering the limited gain of knowledge (3). These methods allow for high resolution characterization of the residues located within the epitope-paratope surface. They fail, however, to provide information on the impact of mutations on this interface. Approaches with higher throughput capabilities, such as peptide arrays and alanine scanning, often fail to provide insight into both three-dimensional and linear epitopes with high resolution. Deep mutational scanning (DMS) was first introduced in 2011 and can efficiently scan large libraries that consist of all possible single amino acid mutations (4, 5). This technology links the effect of a genotype to phenotype, permitting rapid inference of conformational and linear epitopes at an unprecedented resolution. Epitope mapping *via* DMS has had broad applications in the development of therapeutics and diagnostics and proved invaluable during the COVID-19 pandemic for rapid and comprehensive mutational surveillance and forecasting (Fig. 1). We believe this will likely supplant existing epitope mapping methods. We will introduce methods for library generation, selection of variants, and deep sequencing to be employed as tools for developing deep mutational screens.

**Figure 1:**
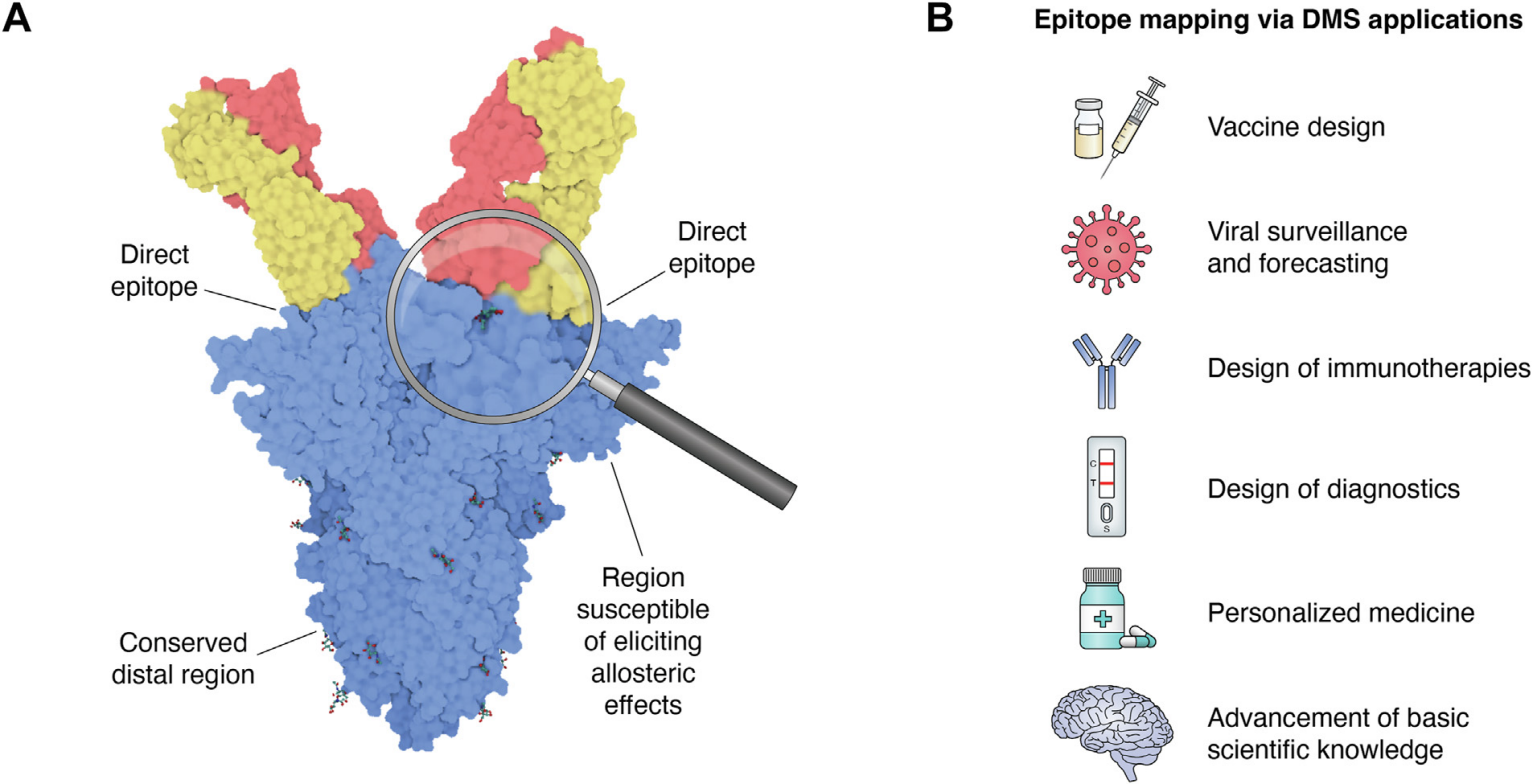
**Epitope mapping *via* DMS applications.***A*, epitope mapping is a technique used to define the structural region responsible for direct protein-protein interactions. Epitope Mapping *via* deep mutational scanning (EM-DMS) can provide a more detailed insight on the effects that individual mutations have on these interactions. Regions that are liable to demonstrate allosteric effects and areas of high conservation are also characterized. The structure featured here can be found in the Protein Database (PDB) under accession code 7U0P (166). *B*, information gained from epitope mapping has broad reaching applications into the rational design of vaccines, therapeutics, diagnostics, personalized medicine, and the advancement of basic scientific knowledge in an unprecedented manner.

## Methods of epitope mapping

### Structure determination

Determining the empirical structure of a protein-protein interaction is regarded as the gold standard in epitope mapping. X-ray crystallography has been the most widely used method providing atomic level resolution (6). The process of generating a crystal that is of high enough quality to diffract can be laborious, making this a low-throughput method. It is important to note that monoclonal antibodies are not good candidates for X-ray crystallography as they are heavily glycosylated, which introduces conformational and chemical heterogeneity, hindering crystallization (6). Thus, crystallization is typically performed using the corresponding fragment antigen-binding region (Fab)-protein complex. Additionally, crystallization can stabilize weak or low frequency interactions that are irrelevant *in vivo*. NMR is another method that can be employed with a highly purified, water-soluble, smaller (<30 kDa) protein (1). NMR has the advantage of not requiring a crystallized protein, although the size restriction often negates this advantage. NMR is typically coupled with isotope labeling, hydrogen-deuterium exchange (HDX), or saturation transfer experiments, allowing this technique to have a relatively higher throughput (1). A relatively new development in the field of structure determination is Cryo-EM. Cryo-EM couples electron microscopy with cryogenically preserved protein samples, keeping the proteins in a near-native state while also reducing damage from the electron beam (7). This technique is more suitable for medium to large proteins or complexes and has a greater probability of determining structures than X-ray crystallography, given optimized conditions. Moreover, advancements in Cryo-EM technology have allowed for resolution comparable with X-ray crystallography, making this an attractive option for the structural determination of an epitope-paratope interaction (8).

Antibody–antigen complexes are rarely conformationally static, and so insight into the conformational flexibility and dynamics of such interactions can provide important additional insight into epitope properties and functions. Such knowledge is key for therapeutic development given that viruses, in many cases, appear to have evolved to secure a degree of structural flexibility in order to evade immune recognition, and even to hide their epitopes. For example, neutralizing antibodies for dengue virus can bind to epitopes that static mapping studies suggest are not exposed on the virus surface, thus suggesting that the virus varies conformationally, causing the epitope to occasionally be exposed (9). Since NMR is typically solution-based, this technique is a prime candidate for such structural dynamics studies and has been applied, for example, in the investigation of high affinity Fabs with structurally diverse interleukin cytokines (10). However, as mentioned above, this technique is typically limited to smaller proteins, thus limiting its widespread use in epitope mapping. HDX coupled with mass spectrometry (HDX-MS) has also been used to study the structural dynamics of conformational epitopes; for example, a study by Liang *et al.* found that antibody recognition of the HIV gp120 envelope glycoproteins correlates inversely with the degree of local dynamics (11). Such a finding suggests that local structural stability could prove to be an important metric for determining likely epitopic sites.

### Alanine scanning

Alanine scanning infers the effects of removing side chains past the β-carbon and identifies important amino acids in an epitope-paratope interaction (12, 13). Alanine scanning is a high-throughput method wherein each residue is mutated to alanine, or glycine when alanine is the WT residue, and functionally characterized (1). This method is labor-intensive as each mutant must be expressed, purified, and assayed separately (14, 15, 16). Furthermore, if an alanine mutation disrupts the proper folding of the protein or induces a conformational change, this may disrupt downstream functional assays, and residues that reside in the epitope may fail to be characterized (16).

### Peptide microarrays

Overlapping synthesized and immobilized peptides on a microplate allows for a high-throughput, relatively affordable, and quick assay making peptide microarrays (PMAs) a popular option for epitope mapping (3). This technique is limited to linear epitopes as only short, non-structured peptide sequences are screened (14). PMAs can be coupled with functional assays such as Pepscan to screen for linear epitopes in a high-throughput manner. Pepscan refers to an ELISA-like assay wherein the immobilized peptides that bind to detection antibodies can be readily identified (14). Inconsistencies in surface chemistry on the microplate can influence these results (3).

### Mass spectrometry coupled with enzymatic hydrolysis or HDX

Mass spectrometry, coupled with enzymatic hydrolysis, identifies linear epitopes by cleaving the antigen into fragments and distinguishing the antibody-bound fragments with MS. This technology can only identify linear epitopes and cannot identify mutations that have an allosteric effect on the interaction. Combined with HDX technology, HDX-MS can map epitopes in a semi high-throughput manner. Regions of a protein that are bound to another molecule will have slower hydrogen-to-deuterium exchange rates relative to unbound proteins (17). HDX-MS, however, is susceptible to false positives since allosteric effects driven by antibody binding may influence residue conformation and solvent accessibility well outside of the epitope (17).

### T-cell-based epitope mapping

While B-cell epitopes are predominantly three-dimensional, T-cell epitopes are linear (1). Linear epitopes may seem relatively straightforward to map, however, T-cells require a peptide-major histocompatibility complex (p-MHC) complex presented by an antigen-presenting cell (APC) to recognize an epitope. This indirect antigen recognition mechanism is weak in nature (1–100 μM) without further stimulation, requiring the development of creative approaches to detect and quantify T-cell-pMHC interactions (3). An early approach relied on p-MHC multimers labeled with either fluorophores or heavy metal ions. However, this approach limited the diversity of T-cell epitopes that could be scanned. Li *et al.* took advantage of the natural mechanism of two cells sharing membrane-associated proteins called trogocytosis (3, 18). Genetically engineered APCs modify the mechanism of trogocytosis to be bidirectional instead of unidirectional, which in turn labels the APC and can be functionally screened *via* FACS. Another creative approach is called Signaling and Antigen-presenting Bifunctional Receptor (SABR), which associates an extracellular p-MHC multimer with an intracellular signal transducer, so that if an interaction occurs, SABR will signal for the transcription and translation of GFP (3, 19).

### *In silico* computational approaches

*In silico* epitope mapping strategies leverage computational modeling, bioinformatics-based, and machine learning methods. Bioinformatic and machine learning methods are data-driven approaches that utilize online databases and repositories for training. Due to the predominance of sequence-based *versus* structure-based datasets, these methods typically predict linear (T-cell and some B-cell) epitopes more effectively than conformational (>90% of B-cell) epitopes (20, 21, 22, 23, 24, 25, 26). For the past 10 years, automated benchmarking tests by the Immune Epitope Database & Tools (IEDB) web resource have consistently shown the suite of immunological “Net” methods to perform best at T-cell epitope prediction (27). The top performing methods within this suite for MHC I and MHC II prediction, respectively, are the NetMHC-4.0 and NetMHCIIpan-4.1 servers, which both use artificial neural networks trained on an integration of binding affinity and mass spectrometry-derived eluted ligand (MS EL) data (28, 29). Clustering algorithms cause the inherently poly-specific (*i.e.* multi-allelic) MS EL data to be logically assigned, preventing their exclusion, thus considerably expanding the training data set size (29). Recently, TFold was introduced, an AlphaFold-based prediction method that claims to outperform these existing methods in MHC I and MHC II peptide prediction (30). Meanwhile, a 2023 benchmarking study of B-cell epitope prediction approaches revealed that only two methods, DiscoTope2 and BEpro, performed better than random across all metrics (31). DiscoTope2 combines structure-derived residue contact counts calculated from surface accessibility measurements with sequence-derived “epitope propensity scores,” trained using nonameric peptide subunits (32, 33). BEpro (formerly PEPITO) retains the propensity scale first introduced for DiscoTope but uses an alternative strategy for determining contact counts which includes information on side chain conformation in addition to solvent accessibility (32, 34, 35). Since this 2023 study, there has been rapid development of epitope mapping methods, primarily driven by the second iteration of the protein structure prediction program, AlphaFold (36). This advance has addressed one of the significant limitations facing conformational epitope prediction, namely the dearth of structural data. Recently, GraphBepi was introduced, a graph-based model for B-cell epitope prediction that incorporates AlphaFold 2-derived structures as input (37). When benchmarked against other leading prediction models, including DiscoTope2, GraphBepi outperformed these competitors in five-sixths metrics (37). DiscoTope itself has been further developed, with its third iteration now including AlphaFold modeling (38). Historically, the performance of AlphaFold-Multimer, AlphaFold’s extension program for protein complexes, with regards to antibody-antigen prediction accuracy has been considered underwhelming, although this situation appears on the cusp of change with the introduction of AlphaFold 3, which has explicitly promised improved results in this area (39, 40, 41, 42, 43).

While these rapid advances have revolutionized structural epitope prediction, care must be taken not to assume that these assignments map precisely onto the functional epitopes. Contact residues do not necessarily contribute favorably to the free energy of binding, and allosteric residues may be essential for binding despite not being located at the contact surface (44). Modeling strategies, which are computational rather than data-driven, have proven useful for functional epitope prediction. Typically, these models optimize protein structures and complexes according to empirical potentials. Scoring strategies vary but a common metric is the Gibbs free energy of binding (45). Antibody-antigen-focused algorithms within the Rosetta suite of programs have largely set the standard for this method of epitope prediction, with recent advances in glycan modeling addressing a previously significant limitation of the technique (46, 47, 48, 49, 50, 51, 52, 53, 54, 55). Another limitation is that modeling is usually slower than machine learning/data-driven strategies. Exploratory campaigns to identify structural epitopes are therefore normally best served by data-driven methods, but if a precise, functional understanding of the epitope is sought, modeling strategies are typically recommended. However, advances in deep learning may also cause modeling strategies for functional epitope mapping to soon be overtaken.

## Deep mutational scanning

### Overview

**Figure 2:**
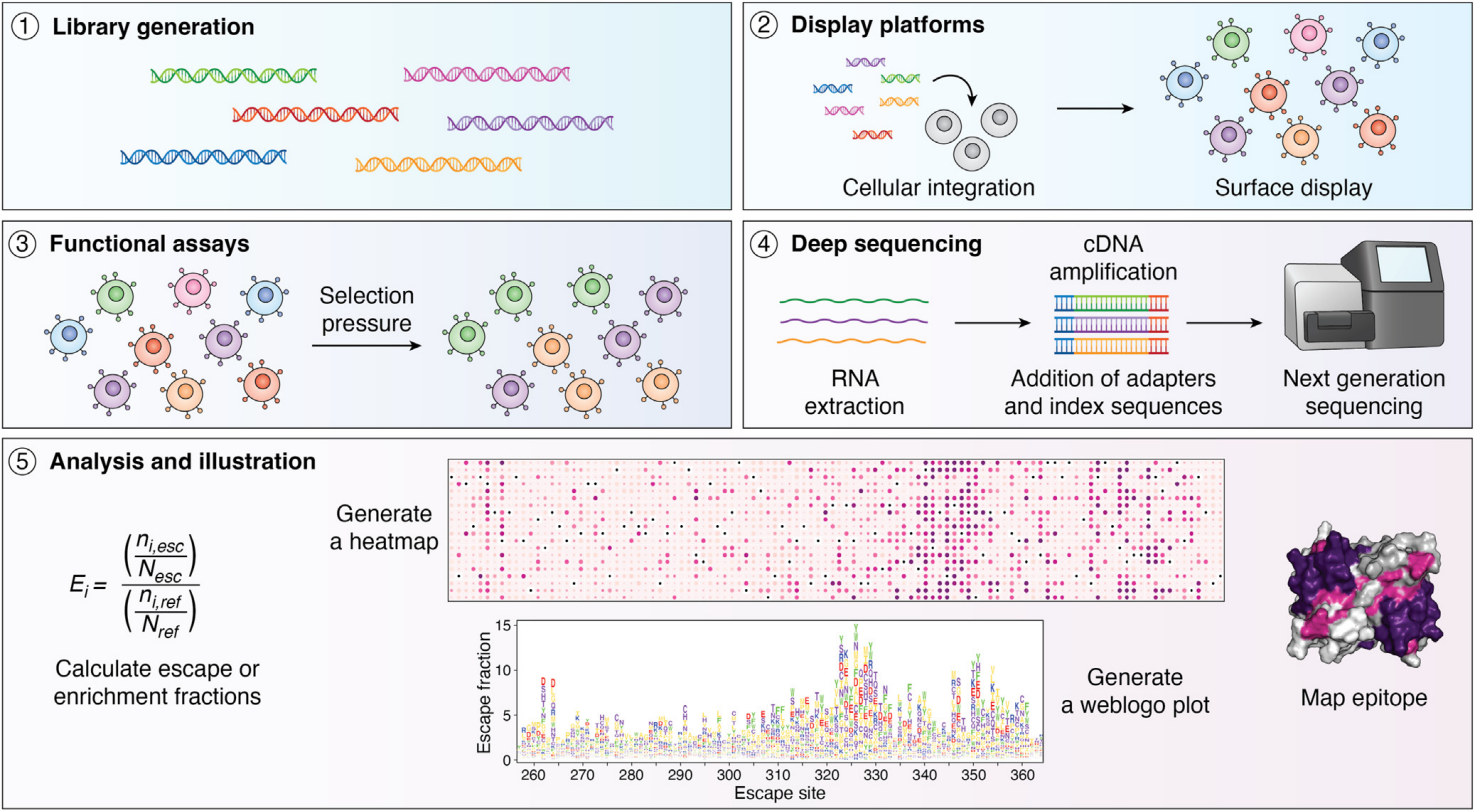
**Workflow of epitope mapping *via* DMS.** The standard workflow begins with the synthesis of a site-saturated mutational library followed by the integration of the library into a display platform to be surface-expressed. Selection pressure is applied to the library cells to sort out the desired population. The selected cells are then prepared for deep sequencing by extracting RNA, generating and amplifying cDNA, appending sequencing adapters and unique molecular identifier sequences. Once the sequencing data is received, algorithmic workflows are performed to link the genotype to phenotype. This information is illustrated with the generation of heatmaps, weblogo plots, and representation on protein surfaces.

Deep mutational scanning (DMS), also known as massively parallel mutagenesis, was first introduced in 2011 (4, 5, 56, 57). DMS screens all possible single amino acid non-synonymous mutations and utilizes deep sequencing technology to identify mutations affected by external pressures. Functional landscape maps link genotype to phenotypic behavior. Traditionally, this technology was employed to identify mutations that affected fitness by performing a cell-growth-based assay, sometimes in the presence of compounds (57). In recent years, DMS has been used to characterize protein-protein interactions and identify mutations that can enhance or escape epitope-paratope interaction. Studies that utilize epitope mapping *via* DMS (EM-DMS) technology all follow a similar experimental procedure—the synthesis of a library, the integration of the library into a display platform, a selection assay of variants, deep sequencing of the selected variants, and data analysis (Fig. 2, Table S1). Functional assays screen for binding capabilities, and deep sequencing measures the abundance of mutations remaining in the population of cells post-selection. The frequency of each mutation in the selected population is compared to the input library to generate an enrichment or escape ratio. Regions of large escape ratios are assumed to be the hotspot of epitope-paratope interaction as epitopes are more susceptible to mutation. Outside the epitope, mutations that elicit an allosteric or conformational effect are identified (Fig. 1*A*) (13). With EM-DMS technology, both conformational and linear epitopes can be inferred in a high-throughput manner. One caveat with this approach is that random mutations are likely to destabilize the protein and should be functionally selected against to delineate against mutations that truly enhance or escape protein-protein interaction (58). The breadth of information generated with an EM-DMS screen can inform the rational design of therapeutics and diagnostics and improve our functional knowledge of protein-protein interactions (Fig. 1*B*).

### Developing a site-saturated library

**Figure 3:**
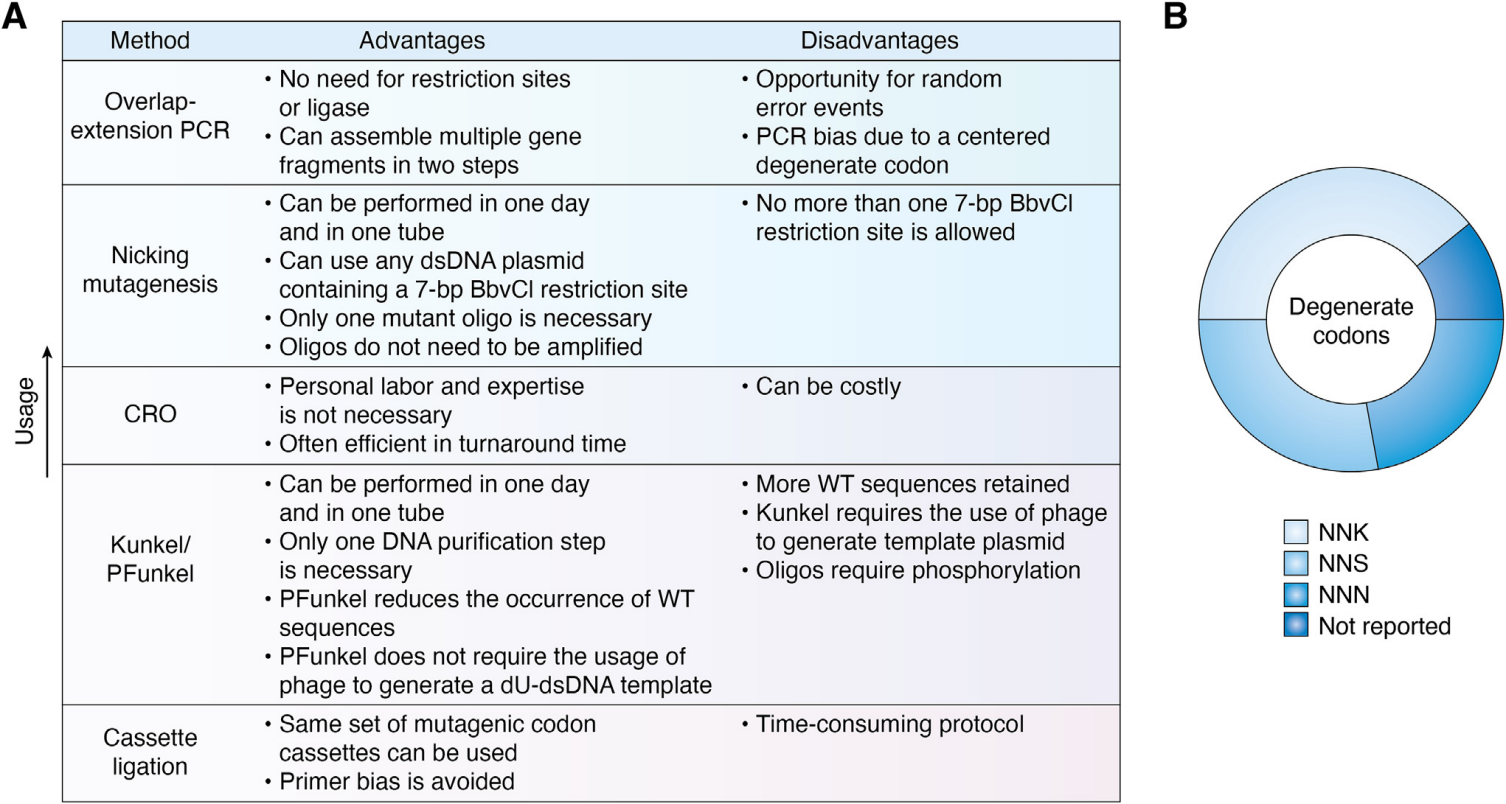
**Synthesis of a site-saturated mutational library.***A*, in the 54 studies that employed epitope mapping *via* DMS, five different synthesis strategies were used to generate a site-saturated mutational (SSM) library, each with its own set advantages and disadvantages which are illustrated here. *B*, different degenerate codons were employed in the generation of SSM libraries. These degenerate codons vary in the representation of the 20 amino acids, frequency of stop codons, and bias towards specific nucleotides.

DMS technology incorporates site-saturation mutagenesis (SSM) libraries into its design to functionally screen all possible single amino acid mutations spanning the length of the desired protein. Degenerate oligonucleotides containing NNN, NNK, or NNS triplets are employed to generate such libraries, where N is A/T/G/C, K is G/T, and S is C/G, all expressed in equimolar ratios. The choice of the degenerate codon dictates the diversity and size of the library. NNN/K/S degeneracy provides coverage of all 20 amino acids, but NNK/S lowers the probability of generating stop codons (59). For example, the NNK triplet covers 32 codons, rather than the 64 covered by NNN, and generates a single stop codon (60). A greater number of residues can be investigated by minimizing the redundancy of codon usage which has made NNK/S degenerate codons the popular choice in designing a SSM library (Fig. 3*B*) (60). Although not observed in the scope of this review, more selective codons exist such as NDT, VHG, or TGG, which lowers the coverage to just 22 codons and no stop codons (60). A transformation step follows library synthesis and amplification to generate an ample amount of plasmid DNA for downstream usage. Appropriate coverage of the entirety of the library is dictated by the number of colony-forming units (CFUs); too little coverage will provide an inadequate representation of variants and too much coverage can provide redundancy and a larger library that requires a greater effort to screen (16). Maes *et al.* observed that at least 10X coverage is required to allow for adequate representation of all variants in the library (16). In synthesis, there is still a chance for more or less than one mutation to appear. In a DMS analysis, variants found with more than one mutation are typically discarded to avoid data convolution. WT sequences, however, can be employed as an internal control. That said, it is also important to reduce the percentage of WT sequences in the library to ensure proper coverage of all possible single amino acid mutations. The destination vector is also an important consideration for library design. The choice of the expression vector should be unique to the display platform and integration technique, such as employing a pLVX mammalian expression vector to generate lentiviral particles. Some of the approaches discussed below require special attention to the restriction sites in the plasmid, such as the required 7-bp BbvCI restriction site in nicking mutagenesis or the requirement with cassette ligation that endogenous SapI remains absent in the recipient plasmid. Moreover, the addition of selection markers can allow for functional selection in downstream experiments. Functional tags such as myc can be used to screen for properly folded protein (61). Discarding variants that lead to improperly folded protein can delineate between a variant that is a true disrupter of a protein-protein interaction and a spurious variant that results in a misfolded protein, which is more likely to occur.

#### Error-prone PCR

Libraries generated by employing error-prone PCR use a low-fidelity polymerase to intentionally incorporate errors during amplification (56). The targeted error rate can be accomplished by modifying the concentration of manganese chloride and dNTPs (56). Starita *et al.* have found that a 2 to 4% error rate is sufficient for this method (62). That said, the disadvantages of using this method are that it results in poor coverage of mutations, and the present mutations are biased toward A/T mutations instead of G/C mutations. For this reason, our review excludes any studies that employed error-prone PCR to generate libraries.

#### Overlap-extension PCR

The most popular approach to library synthesis is overlap-extension PCR (OE-PCR). Used as an affordable and quick means to construct hybrid genes without the need for restriction sites or ligase, OE-PCR assembles multiple gene fragments into a full-length product in two PCR steps (63, 64). OE-PCR often employs four primers: two flanking ones that anneal upstream or downstream of the plasmid and two internal ones that overlap and contain degenerate codons (65). The flanking primers should include unique restriction sites and an adjacent G/C clamp to the 5′ end of each flanking primer to increase subcloning efficiency (65). Furthermore, validation that the flanking primers can amplify the target gene from the plasmid template is recommended to avoid future distress (65). The mutant internal primers should be equal in melting temperature as Dingens *et al.* observed a more uniform rate of mutation when internal degenerate primers of equal melting temperatures were used rather than of equal length (66). Mutations can be targeted anywhere along the gene; however, the flanking primer should contain the degenerate sequence if the targeted codon is within 35 bp of the beginning or end (64, 65). In the first step, flanking primer A and the internal, degenerate primer B produce the gene fragment “AB.” Likewise, the internal degenerate primer C and the flanking primer D produce the gene fragment “CD”. Often, these fragments require gel purification before advancing to the second PCR step. The fragments are then pooled together in equimolar ratios, denatured, and annealed to generate the full-length gene fragment. This full-length fragment is then amplified with the flanking primers A and D. This process is simplified with asymmetric overlap-extension PCR, which relies on the depletion of mutant primers to generate single mutant strands with overlapping 3′ ends (67). Each gene fragment population predominantly consists of these single strands, which are then pooled without the additional purification step to generate full-length, double-stranded mutant DNA in the second PCR step (67). OE-PCR is moderately efficient, affordable, and reliable; however, the chance of random error is increased with each round of PCR, and the degenerate codon that is centered in the internal primer can result in PCR bias due to the thermodynamic mismatch penalty (Fig. 3*A*) (68, 69).

#### Nicking mutagenesis

Another popular approach to generate a SSM library is nicking mutagenesis, first described by Wrenbeck in 2016 (70). This method “nicks” a WT dsDNA plasmid containing a 7-bp BbvCI recognition site with a Nt.BbvCI endonuclease and then exonucleases are used to degrade the nicked strand to generate a ssDNA template. The addition of a pool of degenerate oligos synthesizes the mutant single strands. The second strand on the mutant DNA is synthesized following nicking and digestion of the WT template, thus generating a dsDNA mutant plasmid (70). This method can be performed in 1 day with minimal hands-on labor using any plasmid dsDNA, as long as it contains a 7-bp BbvCI restriction site (Fig. 3*A*). If a plasmid contains more than one BbvCI site, then one must ensure that each site is in the same orientation to avoid nicking in untargeted areas. Furthermore, while only one mutant oligo is necessary for this method, each mutant oligo must undergo 5′ phosphorylation (56). Very little primer is necessary, meaning that oligo pools do not require amplification prior to beginning this procedure (71). For this reason, nicking mutagenesis is described as a “single-pot” reaction, making it apparent why this is a popular technique (70).

#### Cassette ligation

Mutagenic codon cassette ligation was first introduced in 1994 by Kegler-Ebo *et al.* and is employed to incorporate a library of mutations into a specific DNA sequence (72). The cassette contains a three base-pair direct terminal repeat and two SapI recognition sequences, a restriction endonuclease that cleaves outside its recognition sequence (72, 73). The target molecule has a double-stranded blunt break at the site that is targeted for mutagenesis (72, 73). The cassette ligates to the target molecule, and then SapI is digested, leaving a three base overhang that generates the intended substitution mutation (72, 73). The linear, blunt-ended WT template must not contain any endogenous SapI cleavage sites (72, 73). The blunt ends of the template must also flank the position of the targeted codon for substitution mutations (72). This method effectively eliminates the parental plasmid and only restores protein function by complementing the excised area (Fig. 3*A*) (74). Furthermore, without the necessity of annealing primers, bias towards primers with similar sequences to the WT codon is avoided and results in a library with higher diversity (74). The advantage of this approach is that when the target molecule is generated, the same set of mutagenic codon cassettes can be used for mutagenesis, allowing for the introduction of all possible single codon mutations without additional expense (72). The exception, however, is when the generation of blunt ends at the target site is needed (72). This protocol is more time-consuming than other protocols, typically requiring 5 days to complete (73).

#### Kunkel/PFunkel

Both Kunkel and PFunkel mutagenesis strategies employ a phage-derived, single-stranded uracil-containing circular plasmid template (dU-ssDNA), which degenerate oligos can anneal to and generate the mutated double-stranded DNA (75, 76). Kunkel mutagenesis, however, has been reported to retain 30 to 50% of WT sequences (76). PFunkel offers a solution that reduces the occurrence of WT sequences and offers an approach to generate a dU-dsDNA template without phage (76). This dU-dsDNA undergoes thermocycling to anneal phosphorylated degenerate oligos and synthesizes the mutant strand (76). After synthesis of the mutant strand, the uracil-containing plasmid is degraded through selective enzymatic degradation, and a double-stranded mutant DNA is generated with the reverse oligonucleotide (60, 76). Phosphorylated oligos are added in a low oligo:template ratio to reduce the potential of generating multiple mutations (76). The entire PFunkel mutagenesis protocol can be performed in 1 day and one tube, excluding the phosphorylation of the degenerate oligos (Fig. 3*A*) (76). Because there are no separate steps in separate tubes, DNA purification is not necessary until the completion of the protocol (76). One of the most significant advantages of this protocol is therefore that it does not require laborious library construction for every residue, as a comprehensive library can be generated from a single tube in a single reaction (76).

## Expression platforms

### Yeast

**Figure 4:**
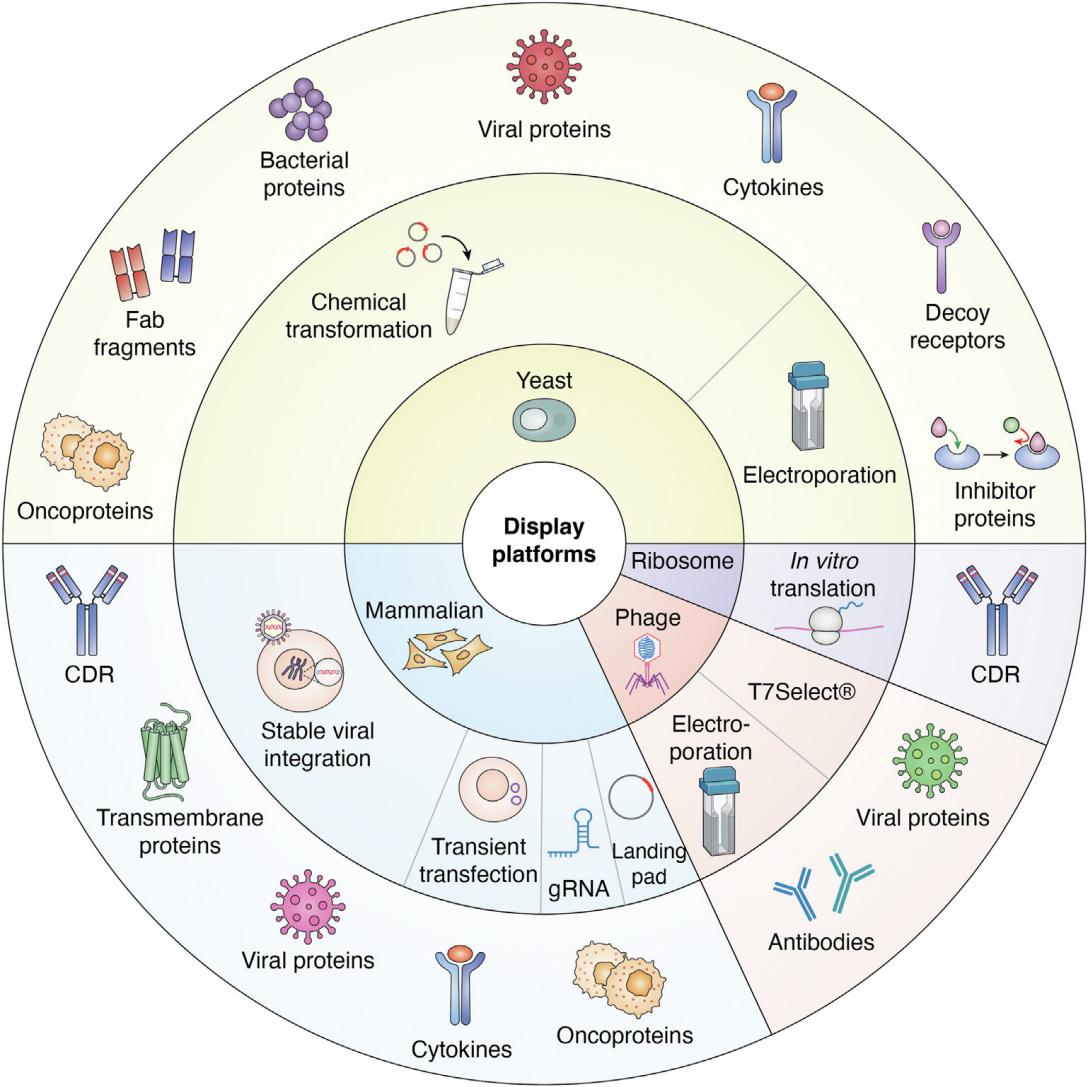
**Display platforms.** All 54 studies which employed DMS for epitope mapping purposes are represented in this circular diagram. The degree to which each display platform or integration method was used is represented by the proportion of each area relative to the entire circle. The inner most circle represents the four display platforms and their respective popularity. The middle circle represents the methods of integration into the display platform of the corresponding color and their respective popularity. The outer circle represents the diversity of proteins that were surface-displayed within each platform of corresponding color.

Yeast expression platforms have been a popular option for DMS as they permit eukaryotic post-translational modifications (PTMs), have similar secretion systems to mammalian cells, and support the proper folding of complex oligomers (Fig. 4) (3). Large libraries with 10^8^-10^9^ variants can be expressed; however, this diversity is still magnitudes smaller than other platforms such as phage or bacterial displays (3, 60). Screening glycan-dependent protein-protein interactions might not be ideal as yeast does not express a terminally sialylated N-glycan that is found on many human membrane proteins (13). Other disadvantages are lower transformation efficiencies and the ability for multiple copies of the protein to be surface expressed (3). Using a low-copy plasmid ensures that each cell is transformed with only one or two copies of the target gene which reduces variation in the amount of surface expressed protein on each yeast cell (62). Thus, the effect of a variant will not be masked by differential protein abundance, enabling cleaner functional selection. Most often, transformation occurs through the LiAc/SS carrier DNA/PEG chemical transformation method (77). In this protocol, the use of single-stranded DNA increases the transformation efficiency (77). Furthermore, the combination of LiAc and PEG stimulates efficient plasmid uptake (78). A lower concentration of plasmid DNA is used in this protocol to limit the opportunity for multiple plasmids to be transformed into a cell (77). Other studies have opted for electroporation to facilitate transformation. Benatuil *et al.* describes a protocol capable of generating libraries up to 10^10^ in size by pretreating the yeast cells with LiAc and DTT and then electroporating a linearized vector in a buffer consisting of CaCl_2_ and sorbitol (79).

### Mammalian

Mammalian expression platforms are a popular choice for membrane-expressed proteins that are decorated with complex glycans as this platform preserves the natural glycosylation patterns of proteins (Fig. 4) (60). That said, it can be challenging to generate a library-containing cell line, especially one that is stable. Transfection efficiencies are often low, and the library diversity is restricted to a range of 10^5^-10^7^ variants (3, 60). Stable cell lines are frequently generated through viral-mediated transduction, although landing pads and CRISPR are other attractive options. Viral-mediated stable cell line generation stably integrates one mutant per cell if transducing at a low multiplicity of infection (MOI) (16). However, it does run the risk of substantial recombination due to the pseudo-diploid nature of retroviruses (16, 80). The inclusion of a non-integrating carrier plasmid that is co-transfected at a greater concentration than library DNA is thought to be able to limit the occurrence of recombination or multiple integrations but will greatly decrease viral titer (81, 82). Alterations to endogenous gene activity can arise from lentiviral transduction (16). Landing pad recombinase systems provide the advantage of only one recombination event occurring (16). There is also the added benefit of no restriction on the size of the transgenic payload (80). Matreyek *et al.* describes their approach in utilizing the serine recombinase, BxbI (80). Serine recombinases are promising candidates for these systems as they only permit one recombination event while excluding the possibility of reversal when directionality factors are not provided (80). The human genome does not contain Bxb1 recombination sites, a factor that allows for specific transgene integration at a defined locus (80). CRISPR is another option to ensure stable integration at a specific subcellular location (16). Transient transfection of library DNA has also been used to express library variants, though this is not a stable integration and can suffer from variability in transfection efficiency. Co-transfection with an empty plasmid with at least two times the amount of library DNA can greatly improve the odds of a single variant being transfected into a single cell, similar to the co-transfection of a non-integrating carrier plasmid in lentiviral integration (83, 84).

### Bacteriophage

While yeast and mammalian expression platforms are limited in their diversity, phage expression platforms can theoretically be up to 10^11^ variants in size (3). This large library might be difficult to screen in other platforms, however, the small size of bacteriophages allows for rapid functional screening (85). Two kinds of phage, T7 and M13, are popular for library display. One of the main differences is that M13 phage will not lyse the host cell, while T7 will lyse post-replication (1, 3). T7 phage is also more efficient in displaying libraries. For these reasons, T7 phage has become a popular choice for this platform (Fig. 4) (3). While the depth of diversity is certainly an advantage with this platform, bacteriophages lack the ability to express large proteins, may not preserve native conformations and oligomeric states, and do not preserve the same PTMs or glycosylation patterns as in the mammalian or yeast display platforms (3).

### Ribosome/mRNA

Ribosome displays allow for functional evaluation of libraries with more than 10^12^ variants (Fig. 4) (60). The *in vitro* transcription of mRNA that lacks a stop codon allows for the protein to be tethered to the stalled ribosome (60). Ribosome display can be adapted to eukaryotic expression systems to allow for PTMs and is relatively quick in identifying variants of interest since cell culture is not required. The stability of this platform is somewhat delicate given the involvement of mRNA and the protein target must fold as a monomer, reducing this platform’s utility for heterooligomer or membrane proteins.

## Functional assays

In the second step of the EM-DMS workflow, functional assays are employed to select for mutations of interest and exclude mutations that result in improperly folded protein (Fig. 2).

### FACS/MACS

**Figure 5:**
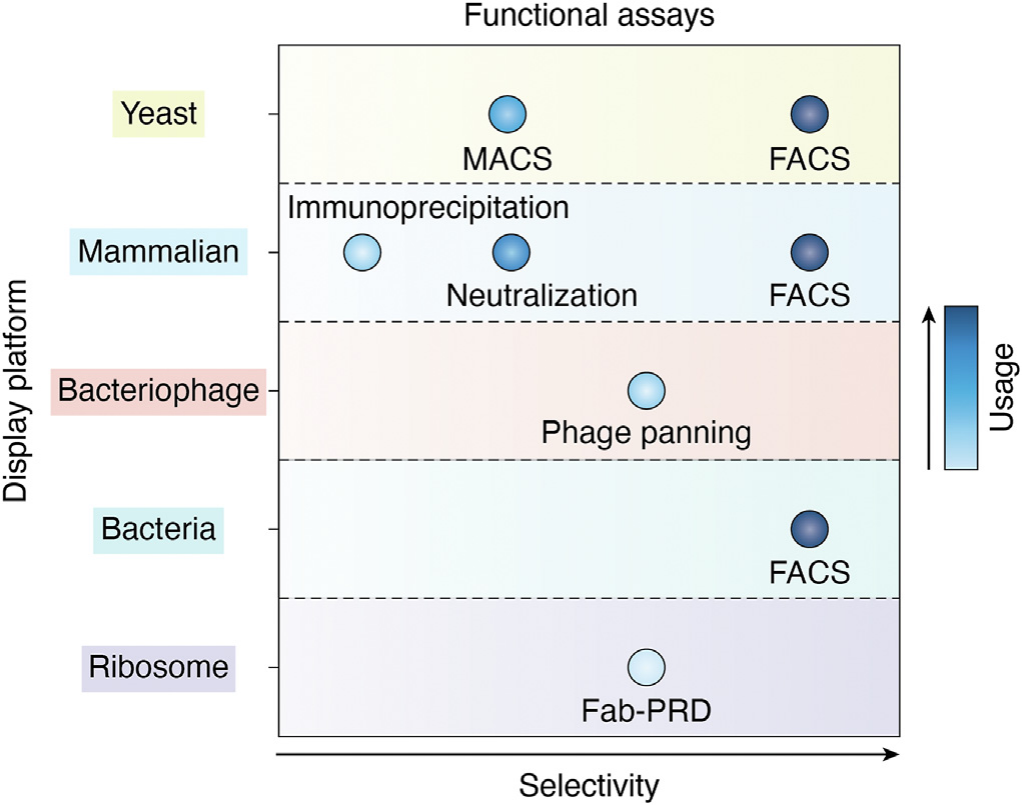
**Functional assays.** The six different functional assays performed in the 54 studies that employed DMS for epitope mapping purposes are represented in this graph. The level of selective pressure that an assay is able to apply to the library cells is represented on the X-axis. The Y-axis is split by the five different display platforms. The usage of each assay is represented by the color of the circle.

Most often, functional screening occurs through FACS experiments wherein the surface-expressed protein is incubated with the targeted binding partner, and mutations that either enhance or escape interaction can be identified and physically separated from the input population (Fig. 5). As a note, it is helpful to determine the apparent dissociation constant (K_D, app_) or the EC90 of the input population before this experiment occurs, which aids in deciding what concentration of the targeted binding partner should be used to incubate with the cell surface-expressed protein. Typically, in an EM-DMS FACS screen, the top or bottom 5 to 15% of binders will be sorted. This narrows the population down to only the tightest or weakest binders. Some opt for a three- or four-way sort to separate cells based on a range of fluorescent signals rather than exclusively sorting for the weaker or tighter binding cells. Adams *et al.* first described a variation to this four-way sorting approach called TITE-seq (86). In this experiment, cell surface-expressed proteins are incubated with a fluorescently labeled binding partner at a cascade of concentrations (86). The cells are then sorted into four bins (86). A negative control is used to set the gate with the lowest signal, denoted as the first bin, and the cells incubated with the highest concentration are used to set the gate with the highest signal, or the fourth bin (86). These gates are not adjusted throughout the experiment (86). With decreasing concentration, the distribution of cells will shift from the fourth bin in the higher signal range to the first bin in the lower signal range (86). This shift of cells enables the apparent dissociation constant (K_D, app_) to be determined for all possible single-point mutations (86). This data is especially useful in affinity engineering when developing therapeutics with rational design. Magnetic-activated cell sorting (MACS) is a variation of FACS that requires magnetic beads to isolate cells of interest. This approach avoids the time cost of FACS, and no instrument is needed. However, MACS does limit the ability to target a population of cells as stringently as what FACS accomplishes with gating strategies (Fig. 5).

### Neutralization/competition assays

Neutralization assays are a popular choice in studies that investigate the interaction between viral proteins and antibodies (Fig. 5). Viral libraries are generated by transfecting mammalian cells, collecting the supernatant, incubating with antibody, and infecting cells once more. The RNA from cells is then isolated to identify mutants that evade antibody neutralization, which can be used to infer the epitope. This assay, however, only identifies the epitopes of antibodies that neutralize viral antigen and is not intended to define the antibodies that mediate viral control through non-neutralizing means (87).

Likewise, understanding how mutations might confer a greater replication fitness or abrogate replication in the presence of antibodies allows for the understanding of viral tolerance and adaptation to mutations. Competition assays identify such mutations with *in vitro* cell culture. Duenas-Decamp *et al.* incubated viral libraries with peripheral blood mononuclear cells (PBMC) to investigate binding between the HIV-1 envelope trimer and the CD4 binding loop (88). The supernatant was then collected at different time points and processed for sequencing. Mutations found to still replicate at later time points than observed for the WT virus were defined to confer a greater fitness (88).

### Phage panning

This approach was employed by Koenig *et al.* to identify mutations conferring high affinity to their phage-displayed anti-VEGF antibody to accomplish *in vitro* antibody maturation (89, 90). Koenig *et al.* conducted phage panning by first selecting against structurally unstable mutations using immobilized protein A, protein L, or anti-gD on an ELISA plate followed by incubation with antigen, elution of bound phage, amplification of selected phage in *E*. *coli*, and then two more subsequent rounds of selection with decreasing antigen concentration (89). They identified mutations adjacent to the framework region of the antibody that increased structural stability and antigen affinity. Multiple selection rounds are necessary with this method to exclude mutations of weaker affinity (Fig. 5).

### Immunoprecipitation

Garrett *et al.* employed immunoprecipitation to isolate phage mutants that complexed with antibody (87). Similar to MACS, the non-binder and binder variants are magnetically separated. Unlike phage panning, this was not performed with multiple rounds of decreasing antigen concentration. While this reduces the time cost of the assay, it does not narrow down the population to the tightest binders (Fig. 5). Nonetheless, they were able to define and map the epitope.

### Fab-PRD

The protein synthesis using recombinant elements (PURE) ribosome display in a single-chain Fab format (Fab-PRD) is a great option when employing a ribosome display platform. In this approach, library DNA undergoes *in vitro* transcription and translation with the help of the PURE system (91). The mRNA-ribosome-polypeptide ternary complex then undergoes affinity selection with a Fab fragment. RT-PCR generates dsDNA, which is used for subsequent rounds of affinity selection (Fig. 5). This approach identifies mutations that retain high affinity to Fab fragments but is subject to noise as multiple rounds of cloning could lead to recombination events. Fujino *et al.* employed this approach for *in vitro* affinity maturation of a Fab targeting tumor necrosis factor-alpha receptor (TNFαR) (92). With just seven amino acid substitutions, they achieved a 2110X improvement in affinity.

## Deep sequencing

In general, the preparation for sequencing is similar across all EM-DMS studies. After functional assays are performed, RNA or plasmid DNA is extracted, RNA is reversed transcribed, and the region of interest is amplified with PCR (Fig. 2). Amplification bias and errors can be reduced by using high-fidelity polymerases and decreasing the number of PCR amplification cycles (16). In amplification, adding sequencing adaptors and unique molecular identifier (UMI) index sequences allows the samples to be pooled for multiplexing. The choice of sequencing platform is often dependent on the size constraint of each platform; for example, Illumina MiSeq has a read length constraint of 2 × 300 bp, where a variable region of 300 bp can undergo paired-end sequencing, or a variable region of 600 bp can undergo single-end sequencing (16, 93). Paired-end sequencing improves confidence in sequencing results by generating double the coverage of the area sequenced. Because of this read length constraint, some have opted to tile their large genes into multiple libraries to allow for full coverage of their gene in sequencing. Others have opted for an 8nt-30 nt barcoded library, which requires long read sequencing before integration into a display platform. Typically, PacBio or Nanopore technology are used to associate the unique barcodes with its corresponding variant, permitting the generation of a look-up table that can be referenced in the future for analysis (16, 93). Barcoding strategies require sequencing only the short barcodes, reducing sequencing cost and turnaround time (62). The addition of multiple barcodes per variant generates a set of internal replicates that is useful for statistical analysis and increased confidence for functional annotation of variants.

## Analysis

Upon receipt of the sequencing data, multiple steps are applied to convert this information into a phenotype-related score with appropriate error quantification (Fig. 2). Numerous strategies have been developed to achieve these aims, all of which broadly incorporate the following steps.

1. Parse the sequences (or barcodes).
2. Filter out any low-quality sequences.

(2∗. If barcoded, associate these with the appropriate variants.)

3. Count the variants.
4. Normalize the variant counts.
5. Quantify the frequency change of the variants (*i.e.* generate a score).

(5∗. If there are replicates, merge the scores and quantify random errors.)

A successful analysis workflow must control potential errors from emerging at each step, and so increasingly sophisticated frameworks have been developed to minimize such errors, provide better quantification, and improve standardization across experimental platforms.

Enrich and EMPIRIC were among the first to emerge as leading DMS analysis platforms, which calculated scores using simple ratios of the variant frequencies before and after selection, and quantified error using a Poisson *t* test (Enrich) or a two-sided Student’s *t* test (EMPIRIC) (5, 94). Although intuitive and easily calculated, simple ratio-based scoring is prone to sampling error (95). Even when pseudocounts are applied (which exclude ratios of zero or infinity), ratios arising from finite counts introduce statistical biases, with the bias increasing when the count frequency is low, and which propagates through subsequent analyses (96, 97, 98). To avoid this issue, the software package, dms_tools, implements a Bayesian approach for inferring site-specific preferences (99, 100). Prior estimates for π_*s*,*r*_, which denotes the preference of site *s* for residue *r*, are made, with the assumption that the preferences for all possible identities (*i.e.* residues) are equal and that the mutation and error rates of each site are equal to the library averages. A Markov chain Monte Carlo simulation is then run to calculate the posterior mean of the preferences. This approach is essentially guaranteed to perform as well or better than the simple ratio-based approach but at high sequencing depth the increased complexity (and associated computational runtimes) may not be worth the marginal benefit in accuracy (100). Enrich2, meanwhile, retains the ratio-based approach (albeit using log ratios) with Poisson assumptions for reference-output experiments (95). An important advance made with Enrich2 is in the analysis of experiments exceeding two sequenced populations (such as in time-courses). For such analyses, linear regression-based scoring is typically used. A complicating factor with this method of scoring encompasses the appropriate normalization of data. Prior to Enrich2, normalization to wild-type frequency was the preferred approach. Some approaches simply subtract the wild-type score from each respective variant score, not accounting for potential wild-type frequency non-linearity (101). A more sophisticated approach is to normalize each variant score to wild type at every time point (5, 102, 103, 104). This approach, however, cannot be accurately implemented under the following circumstances: when the wild-type sequence is unknown; when the effect of the wild type is subject to high levels of error, giving large outliers; and when read depth is low (105). To overcome these possible issues, Enrich2 can normalize with respect to the number of reads instead of the wild-type frequency. To mitigate the problem of different time points having different numbers of reads plus time points with low coverage being more prone to sampling errors, Enrich2 also offers weighted regression (95). DiMSum, introduced in 2020, focuses on improving error quantification for two-point experiments (106). As such, variant scoring is achieved using the natural logarithm of the ratio between output and input sequencing counts, similar to Enrich2 two-point assays. However, the developers of DiMSum argued that the assumption made in Enrich2 that variant frequencies are Poisson distributed typically fails to control type-I errors (*i.e.* false positives), especially for over-disperse datasets. As such, DiMSum introduces novel additive and multiplicative modifier terms to mitigate over-dispersion of the sequencing counts. One useful feature of these modifier terms is that they typically arise at different points within the experimental workflow. Therefore, these terms can help researchers pinpoint poorly designed aspects of their experimental setup, with troubleshooting advice provided in the original DiMSum publication (106).

DMS analysis methods are advancing rapidly, producing alternatives to the more established strategies described earlier. The R package, mutscan, was recently published, with its variant scores strongly correlating to those produced by Enrich2 and DiMSum using a fraction of the computational cost (107). Further advantages of mutscan are its modularity and ease of use. However, there is concern that the pre-existing packages which mutscan is built out from, edgeR and limma-voom, were originally designed for RNA-seq data, which typically shows greater consistency between replicates than DMS (108, 109, 110, 111). Furthermore, the parsing strategies employed by mutscan appear to be less flexible, resulting in lower counts (107). Rosace is an alternative framework recently introduced (111), which incorporates residue position information, with the understanding that residue position carries information on likely functional effects. In a comparative analysis, the developers of Rosace demonstrated that their method had higher sensitivity for positive control variants while controlling for type-I errors better than Enrich2, DiMSum, and mutscan could achieve (111). A recent, unreviewed article introduces popDMS, which uses statistical methods from population genetics in place of either ratio-based or regression methods to calculate scores (112). This approach views the round(s) of phenotypic selection achieved by DMS as analogous to the rounds of reproduction in natural populations and gives higher consistency between replicates than alternative methods (112).

Visualization of DMS data is typically achieved by heatmaps, weblogo plots, or representation on protein surfaces (Fig. 2) (113). Heatmaps are typically represented by the residue position on the *x*-axis and the amino acid variant on the *y*-axis, creating a grid that is then color-coded according to the functional score. This style of representation provides a simple, visual scheme for identifying the effects of individual variants and, if present, linear epitopes. Weblogo plots are also typically represented by residue position on the *x*-axis, with the *y*-axis being a measure of amino acid preference, where the height of the single-letter amino acid code corresponds to its relative abundance. Through summation or averaging, functional per-site scores can be generated from per-variant scores, and, with a color scheme, these results can be mapped onto protein structures. This method of representation therefore allows for easy identification of conformational epitopes.

The rapid growth of DMS and, more broadly, Multiplexed Assay of Variant Effect (MAVE) studies, prompted the creation of MaveDB, an open-source platform for sharing experimental data, which now (as of May 2024) boasts over 2500 datasets (114). It has also become common practice for DMS analysis frameworks to be made freely available, with the developers of Enrich2, dms_tools, DiMSum, Rosace, mutscan, and popDMS all providing public GitHub repositories. Additionally, the Atlas of Variant Effects Alliance (https://www.varianteffect.org) has been established to assist collaboration between, and increase the influence of, mutational scanning research communities.

## Epitope mapping applications

### Immunotherapies

The output of EM-DMS provides a breadth of information capable of identifying mutations that enhance or disrupt the epitope–paratope interaction. This information can guide the rational design of therapeutics (Fig. 1*B*). A dual affinity Fab against both VEGF/Ang2 was optimized through EM-DMS by defining the two different interactions that occurred within a common binding site (90, 115). Therapeutic antibodies were modified to be absent of CD4 T cell epitopes, as they were found to be the culprit in producing anti-drug antibodies, by identifying mutations that reduce the binding of HLA class II molecules but still preserve the biological function of the therapeutic antibody (116). Additionally, EM-DMS has been employed for the *in vitro* maturation of antibodies and has identified enhancing mutations not identified with other means of *in vitro* maturation. In one case, this resulted in a 2110x increase in affinity (92). In another, the ability to identify core and allosteric mutations within a rare SARS-CoV-2 and SARS-CoV-1 shared epitope allowed for the engineering of a cross-reactive nanobody with subnanomolar affinity (117). EM-DMS has also been employed to define CAR-T cell epitopes (118, 119). Defining the epitope of a commonly used antigen-recognition domain in anti-CD19 CARs, used to treat B-cell malignancies, revealed a conformational epitope that matched clinical data from patients who had relapsed with altered forms of CD19 (118). Knowledge of sequence-function relationships elucidated an otherwise unknown mechanism of therapeutic resistance (118).

### Vaccine design

The COVID-19 mRNA vaccine was developed at an unprecedented speed, yet it still took a year to develop. The Coalition for Epidemic Preparedness Innovations (CEPI) has proposed a lofty goal of generating the next vaccine within 100 days of “disease X” identification (120). The hope is that scientists can gather information on several viral genera or families to use as an exemplar for vaccine design. EM-DMS could greatly aid in achieving this goal by identifying protein hotspots, mechanisms of escape, and regions that can provide cross-protection amongst species (Fig. 1*B*). Cohen *et al.* developed a mosaic nanoparticle that displays the sarbecovirus spike receptor-binding domains (RBDs) of SARS-CoV-2 and seven animal sarbecoviruses (121). This vaccine platform was shown to protect against variants of SARS-CoV-2 and SARS-CoV challenges, supporting the idea that this platform could potentially protect against future SARS-CoV-2 variants or spillover into other sarbecoviruses (121). They employed EM-DMS to evaluate the viral escape and binding preferences to polyclonal sera, which was collected after the immunization of their mosaic-8 nanoparticles (121). EM-DMS has also been employed to aid in the design of vaccines that elicit broadly neutralizing effects against the HIV envelope trimer and cross-protective monoclonal antibodies against needle tip proteins found in *Salmonella* and *Shigella* (122, 123, 124).

### Diagnostics

Frank *et al.* employed EM-DMS to identify the sites of epitope-paratope interaction for antibodies found in COVID-19 rapid antigen tests (RATs) (Fig. 1*B*) (61). The rapid rise of SARS-CoV-2 variants induced concerns surrounding the ability of the RATs to detect the most current circulating viral variants and accurately diagnose infected individuals. A few variables in a RAT could lead to improper diagnosis, such as user error and novel variants escaping detection by the diagnostic antibody. For this reason, EM-DMS was an ideal candidate for screening the efficacy of antibodies found in RATs as it provided a clear understanding of which mutation posed a risk in evading the epitope-paratope interaction. Most antibodies used in RATs detect the SARS-CoV-2 nucleocapsid protein, which is present at much higher levels as compared to the SARS-CoV-2 glycoprotein and reaches detectable levels early in infection. Frank *et al.* employed a mammalian surface display platform of SARS-CoV-2 nucleocapsid to identify mutations that were susceptible of escaping antibody recognition (61). The data generated from this EM-DMS screen can be cross-referenced with the known mutations found within circulation to estimate the risk that variants impose on the performance of the RAT result. Conversely, mutations that enhance the interaction are also identified. These mutations are likely to be found distal to the epitope and be areas of high mutational tolerance.

### Viral surveillance and forecasting

The vast majority of EM-DMS studies thus far have evaluated viral escape mechanisms. It was then later employed to monitor viral escape from convalescent plasma or pre- and post-immunization sera. This allowed for the forecasting of and preparation against variants of concern that were liable to be seen in circulation (Fig. 1*B*).

## Limitations and future advances needed

DMS is usually limited to generating a genotype-phenotype landscape with single amino acid substitutions only, as current designs typically preclude an evaluation of the synergistic effects of multiple mutations. Enormous library sizes would be challenging to integrate into a display platform, screen, sequence, and analyze the data. This could be mitigated by incorporating a tiling strategy to cover the entire gene with multi-site mutations, although this would still be laborious and expensive. Tiling and barcode strategies circumvent difficulties with long-read sequencing technology; however, advancements to expand the read length capabilities are much needed. Advancements in automation technology could prove useful in library synthesis and sample preparation for sequencing, potentially improving efficiency and reducing the cost of labor and time. Moreover, machine learning could be leveraged to predict the functional scores for higher-order, multi-site mutations using DMS data, structural information, and evolutionary information to inform its prediction (125, 126). All means of epitope mapping, minus *in silico* methods, are *in vitro* which means that protein-protein interaction may behave differently *in vivo*. As with all *in vitro* studies, validating the results *in vivo* is recommended when applicable. With more EM-DMS studies being published each year, establishing a clear and standardized reporting framework will be important for effective communication and reproducibility. Claussnitzer *et al.* provides a guideline to reporting MAVEs such as EM-DMS (127).

The breadth of information gained from EM-DMS presents an opportunity to make a significant impact in the advancement of personalized medicine. For example, the generation of a functional landscape map of on-target resistance mutations that can be referenced when strategizing a patient’s treatment plan could inform the choice of an immunotherapy treatment strategy that would confer the greatest patient response (128).

## Conclusions

In this review, we have discussed traditional means of epitope mapping and compared them to DMS. While some methods offer empirical structural information, they are low-throughput or are not optimal in characterizing both the discontinuous and linear epitopes. EM-DMS provides a comprehensive analysis of how single non-synonymous mutations affect epitope-paratope interaction. This information identifies both discontinuous and linear epitopes while also identifying mutations that have an allosteric effect on interaction (Fig. 1*A*). We have also provided insight into methods capable of constructing libraries, integrating libraries into display platforms, functional selection of variants, and deep sequencing preparation and analysis. EM-DMS has been implicated in broad applications such as the rational design of immunotherapies, vaccines, and decoy receptors (Fig. 1*B*). Mutations that were found to improve the design of these therapeutics were often overlooked in other methods. EM-DMS proved invaluable during the COVID-19 pandemic by monitoring and forecasting the mutational landscape of SARS-CoV-2. EM-DMS is revolutionizing the field of epitope mapping with its ability to identify epitopes rapidly and will likely supplant existing epitope mapping methods.

## Data availability

All supporting data are provided within the manuscript, supplementary data and supplementary tables.

## Supporting information

This article contains supporting information (61, 66, 83, 84, 87, 88, 89, 90, 92, 115, 116, 117, 118, 119, 121, 122, 123, 124, 129, 130, 131, 132, 133, 134, 135, 136, 137, 138, 139, 140, 141, 142, 143, 144, 145, 146, 147, 148, 149, 150, 151, 152, 153, 154, 155, 156, 157, 158, 159, 160, 161, 162, 163, 164, 165).

## Conflict of interest

The authors declare that they have no conflicts of interest with the contents of this article.
